# Idiopathic vulvodynia - a psychosomatic disease? A cross-sectional single-center study

**DOI:** 10.1186/s12905-026-04387-1

**Published:** 2026-03-11

**Authors:** Ronja Fierz, Cornelia Betschart, Vera Berger, Gian-Piero Ghisu, Brigitte Leeners

**Affiliations:** 1 Medical School, University of Zürich, Rämistrasse 71, Zürich, 8006 Switzerland; 2 Department of Gynecology, University Hospital of Zürich, Zürich, Switzerland; 3 Department of Reproductive Endocrinology, University Hospital of Zürich, Zürich, Switzerland

**Keywords:** Chronic vulvar pain, Idiopathic vulvodynia, Personality, Sexual abuse, Sexual violence

## Abstract

**Background:**

Idiopathic vulvodynia is a chronic vulvar pain condition that occurs with a high burden of disease and frustratingly low therapeutic success. Psychosomatic factors are discussed to be involved in the onset and presentation of the disease. Sexual abuse has been positively associated with vulvar pain in general; similarly, patients suffering from vulvar pain are discussed to carry certain personality characteristics. Our aim was to examine two psychosomatic aspects: sexual violence/abuse and specific personality traits as associated factors for this particular patient cohort of idiopathic vulvodynia.

**Methods:**

This cross-sectional single-center study recruited 85 previously diagnosed vulvar pain patients (34 idiopathic vulvodynia, 31 eczema and 20 lichen patients) that visited the vulva clinic and 30 asymptomatic women that consulted the contraception or dysplasia consultation clinic at university hospital Zürich. Underage women and patients with vulvar pain due to other somatic diseases were excluded. All participants completed the Big Five Inventory-42 (BFI-42) questionnaire and the 3 vulvar pain groups additionally completed a customised questionnaire regarding vulvar pain symptoms and history of sexual violence/abuse. In the statistical analysis, the idiopathic vulvodynia patients were compared to the other vulvar pain groups in terms of history of sexual violence/abuse, vulvar pain symptom severity and personality traits. For the latter analysis, the asymptomatic control group was also included. Furthermore, an age-adjusted logistic regression was performed as confounder-control in the sexual violence/abuse analysis and Wilcoxon rank-sum tests included in the personality analysis.

**Results:**

In total, 25% of all vulvar pain patients had a history of sexual abuse, with a prevalence of 17.7% in the idiopathic vulvodynia group. Symptom severity results were similar in idiopathic vulvodynia and other vulvar pain patients, whether they had experienced sexual violence/abuse or not. Also, the logistic regression analysis did not find any age-dependent association of sexual violence/abuse history and vulvodynia. Furthermore, no distinctive divergence in personality traits could be detected in idiopathic vulvodynia patients, neither in comparison to genital eczema or lichen patients, nor to control women. A finding that was further emphasised by Wilcoxon rank-sum testing. Median neuroticism scores in idiopathic vulvodynia patients were, however, highest in patients that reported the least or medium symptom severity.

**Conclusions:**

Sexual violence/abuse rates of all vulvar pain groups were higher than the estimated rate in the general female population. However, our findings did not suggest a higher prevalence of history of sexual violence/abuse in the idiopathic vulvodynia patients compared to the other vulvar pain groups and no specific personality profile could be demonstrated in idiopathic vulvodynia patients. Furthermore, the subgroup analyses did not show particularly high rates of sexual violence/abuse history or a distinct personality profile in idiopathic vulvodynia patients with more severe symptoms. Due to limited sample size in the present study, such correlations might, however, only be visible in larger patient cohorts. Also, other psychosomatic factors might be of more relevance in distinguishing the disease of idiopathic vulvodynia from other vulvar pain conditions.

## Background

Vulvodynia is defined as vulvar pain lasting at least 3 months that occurs spontaneously or after provocation and can be accompanied by increased vaginal tension or muscle spasms, indicating vaginismus [1]. Depending on the underlying definition and respective sampling of the participants investigated, 8-19.3% of the global population with female sex assigned at birth (in this study referred to as women) are estimated to be affected by vulvar pain [2–4]. The prevalence of idiopathic vulvodynia, as vulvar pain without identifiable cause [1], is reported to be around 6.5% [5]. Dyspareunia and vaginismus are frequently associated with vulvodynia, as sexual intercourse is considered the main trigger of vulvar pain, followed by significant disruptions of sexual, relational and psychological functions [6–8]. This leads to excessive physiological and psychological burden, which is further exacerbated by family planning matters [9].

The aetiology of vulvar pain is known to be multifactorial, ranging from acute local infections to chronic inflammatory dermatoses such as eczema and lichen [10, 11]. Further contributing factors are nervous system mechanisms, pelvic floor muscle dysfunction, trauma, genetic predispositions and hormonal changes [10–13]. Ultimately, many forms of vulvar pain can be attributed to somatic diseases, although individual symptoms show important variations.

However, although multiple biomedical, neurological, and psychosocial mechanisms have been proposed to be associated with idiopathic vulvodynia, no single cause has been identified, and current perspectives emphasize a multifactorial biopsychosocial model [10]. Lack of developmental understanding makes successful treatment particularly challenging [13]. This forces patients to follow a symptomatic, often prolonged treatment of only limited success, resulting in a particularly high burden of disease with severe multifactorial and often long-term implications [10, 11, 13].

Previous literature suggests a correlation of psychological and psychosomatic factors and gynecological pain disorders, such as vulvodynia and endometriosis [14–16]. Psychosomatic mechanisms are surmised as complex interactions between mind and body with non-physical stressors that might lead to onset of nociplastic pain conditions as much as chronification or exacerbation of existing somatically derived conditions [16, 17]. Depressive or anxiety disorders as well as physical and childhood abuse, adulthood sexual violence and harm avoidance, a typical response to trauma, have been reported to be associated with an increased risk for vulvar pain [15, 18–25]. Furthermore, literature supporting the role of child sexual abuse and/or sexual violence in various medical conditions are accumulating [26–31] and an unfavourable treatment outcome in patients with history of sexual abuse has been affirmed in other pain disorders [32]. Child sexual abuse can roughly be defined as any form of sexual act with a person that is considered a child from developmental and/or legal standpoints and thus, unable to consent to such actions [33]. On the other hand, the term “sexual violence” is used for any kind of sexual action forced on a woman/person without their consent [34]. With the global childhood sexual abuse rate in girls estimated around 25%, and a recent WHO release stating a lifetime risk of sexual or partner violence in women of almost 33% [35, 36], sexual violence and/or abuse may represent an associated factor of particular clinical relevance.

Additionally, specific personality traits seem to be contributing or mediating associated factors in various gynecological areas, such as endometriosis, premenstrual syndrome or sexual functioning [37–39]. As an example, a particularly high disease burden in endometriosis patients showed a personality-dependent variance and neuroticism has been associated with poorer coping in patients suffering from chronic pain [37, 40]; raising the question whether similar associations might be ascertained in idiopathic vulvodynia patients. Furthermore, women with vulvodynia were previously characterized as being cautious and pessimistic [21]. In addition, neuroticism as well as lower levels of agreeableness and extraversion were found to be associated with vulvar pain in general [4, 41].

However, research on idiopathic vulvodynia remains sparse [10]. Studies often do not meticulously differentiate between different types of vulvar pain. Also, potential causes and associated or specific risk factors are not systematically evaluated. Although the available literature supports a correlation between both vulvar pain and abuse and vulvar pain and specific personality traits, a specific association with idiopathic vulvodynia has not yet been examined [18, 21, 22, 42]. Also, no research has yet addressed a potential impact of associated sexual violence/abuse experiences on symptom severity.

This leaves healthcare providers with limited and nonspecific treatment options (no gold-standard treatment guideline for idiopathic vulvodynia) and often leads to frustration in both patients and clinicians. To improve diagnostic quality and related treatment options, further research on idiopathic vulvodynia is required, especially as current research suggests psychosomatic approaches to be promising [43–48].

The aim of the present study was therefore to examine potential correlations between a history of sexual violence/abuse and personality trait dimensions and the distinct population suffering from idiopathic vulvodynia. For this purpose, idiopathic vulvodynia patients were compared to lichen- and eczema-derived vulvar pain patients in terms of history of sexual violence/abuse and vulvar pain symptom severity (pain frequency, duration of pain, and feelings of vaginal tightness). Personality trait dimensions were assessed in idiopathic vulvodynia, eczema and lichen patients as well as in asymptomatic control women, and in a subgroup analysis further evaluated in the vulvar pain groups in regard to symptom severity.

## Methods and Materials

### Study design

This study was conducted as a cross-sectional single-centre study from August 2021 until December 2022 at the tertiary vulva clinic and the Department of Reproductive Endocrinology at the university hospital Zürich (USZ).

### Participants/definitions

**Fig. 1:**
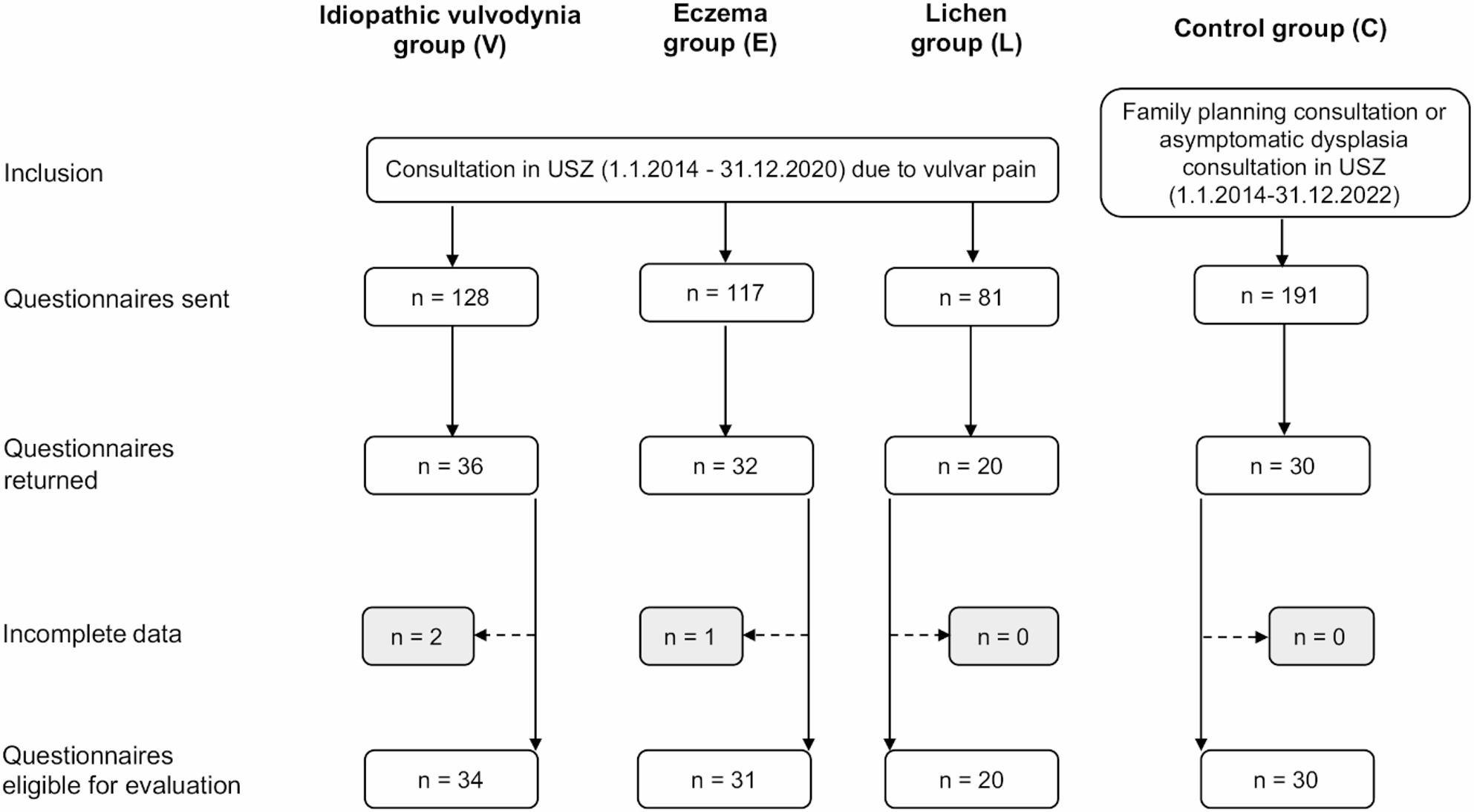
Participant inclusion chart

Vulvar pain patients: All women consulting the vulva clinic at USZ between 1.1.2014 and 31.12.2020 due to vulvar pain were screened for underlying acute and chronic somatic genital diseases, e.g. present/previous urogenital infections, autoimmune diseases, previous local surgical treatment and indication for pudendal neuralgia, overactive bladder or hormonal imbalances, as for example the genitourinary syndrome of menopause. In addition, vulvar pain patients were particularly thoroughly screened for signs of endometriosis, as a frequent disease in the respective patient cohort. Idiopathic vulvodynia patients were diagnosed as such by a specialised gynecologist at the vulva clinic in accordance with the ISSVD guidelines [1]: Recurring vulvar pain for a minimum period of 3 months with positive Q-tip test and (after meticulous medical history research) confirmed absence of local inflammation and history of or current additional symptoms suggesting an underlying somatic disease. Patients presenting with vulvar pain due to carcinoma or acute genital diseases/infections or vulvar pain, which could at least to some extent be explained by somatic reasons, as mentioned above, were strictly excluded as potential study participants. Idiopathic vulvodynia patients were included whether suffering from provoked, spontaneous of mixed vulvar pain. Eczema and lichen patients were diagnosed with the respective disease if they stated vulvar pain, itching and/or burning symptoms for a minimum period of 3 months and presented with either pathognomonic clinical presentation or suggestive clinical appearance followed by positive skin biopsy. Patients diagnosed with idiopathic vulvodynia (V), and those suffering from vulvar pain due to genital eczema (E) or lichen disease (L) were included, provided they stated vulvar pain (Fig. 1). With a given somatic cause in the eczema and lichen groups, two valuable symptomatic control groups were ensured.

As apart from etiological differences, genital lichen sclerosus, ruber and chronicus simplex patients share the same vulvar symptoms with an undisputable somatic cause, dermatologic lichen subtypes were combined in the lichen group. Asymptomatic control group: Women visiting the contraception consultation at USZ between 1.1.2014 and 31.12.2020 without any genital symptoms were asked to support the study. To enlarge the asymptomatic control group (C) due to especially low participation rate in this group, asymptomatic attendees visiting the dysplasia consultation between 01.06.2022–31.12.2022 were also invited to join, on verbal and written request. As the finding (no vulvar pain) was comparable in the two groups recruited in two different consultations, the key variable was equally distributed and therefore adjusted.

In contrast to somatic diseases, psychiatric comorbidities were no exclusion criterion in any participant group and vulvar pain patients were included in all treatment stages. Further inclusion criteria for all groups were a minimum age of 18 years and fluency in German language to ensure reliable questionnaire completion. All in all, 128 idiopathic vulvodynia, 117 eczema and 81 lichen patients as well as 191 asymptomatic women were asked to participate in the study by written request.

### Instruments/questionnaires

Two questionnaires were programmed on the REDCap survey platform: The german Big Five Inventory-42 (BFI-42), a modified version of the BFI-44 originally created by John and Rammstedt in 1991 and validated in people of young, moderate and later age in 2001 [49, 50], and a customised questionnaire on symptoms and treatment outcomes in women with vulvar pathologies previously developed by the research team and published in 2024 [43].

The BFI-42 questionnaire was chosen to examine the 5 main dimensions of character traits in all participant groups. It consists of 42 questions with 7–9 questions regarding each of the 5 dimensions: extraversion (e.g. energetic, talkative), agreeableness (e.g. supportive, considerate), conscientiousness (e.g. reliable, tidy), neuroticism (e.g. anxious, nervous), and openness (e.g. inventive, imaginative) [49].

The genital pain questionnaire contains 37 questions concerning symptom occurrence, pain frequency, duration of pain, and vaginal tightness when having intercourse/using a tampon, as an indication of present vaginismus. Patients were asked about vaginal parity and whether they had endured any physically harmful or emotionally hurtful sexual experiences before onset of vulvar pain. Such experiences were referred to as experiences of sexual violence/abuse in this study. No distinction was made between sexual abuse that happened during childhood or sexual violence experienced in adulthood; however, it was considered essential whether it had occurred before noticing vulvar complaints. Since the latter questionnaire comprises almost exclusively symptom-related questions, it was only used with the vulvar pain groups (V, E and L); therefore, we lacked information on sexual violence/abuse experiences in the asymptomatic control group.

### Study conduct

Customised IDs were generated on the REDCap survey platform and individual study invitations sent by email for the solely online conduction of the survey from August 2021 to December 2022. Via REDCap link all potential participants were informed about the aims and conduction of the study. Following written informed consent, participants of the vulvar pain groups were forwarded first to the genital pain questionnaire and later to the BFI-42 questionnaire. The asymptomatic control group was directed to the BFI-42 questionnaire only. An e-mail reminder was sent after 3–8 weeks to potential participants who had not responded to the first study invitation. Eventually, all women that had filled out at least 95% of the BFI-42 questionnaire were included in the study with a total of 115 participants: 34 idiopathic vulvodynia, 31 eczema and 20 lichen patients as much as 30 asymptomatic control women. The lichen group consisted of 14 lichen sclerosus, 4 lichen simplex chronicus and 2 lichen ruber patients. Data of all the participants’ date of birth, ethnicity/nationality, and education were extracted from electronic medical charts. For the asymptomatic control group, data of vaginal parity was also extracted. This contrasts with the vaginal parity data in vulvar pain patients, as this information was included in the genital pain questionnaire.

#### Sexual abuse analysis

For the sexual violence/abuse analysis two self-reported items of the customised questionnaire were used to differentiate whether the vulvar pain patients had endured any physically harmful or emotionally hurtful sexual experiences before onset of vulvar pain. Such experiences were referred to as a history of sexual violence/abuse in this study. Furthermore, symptom severity was compared in terms of pain frequency, duration of pain and feelings of vaginal tightness in participants with and without history of sexual violence/abuse overall all 3 vulvar pain groups (idiopathic vulvodynia, eczema and lichen patients).

#### Personality trait analysis

To enable comparison between the vulvar pain groups and asymptomatic women, the asymptomatic control group was integrated into this analysis. The 42 self-reported BFI-42 questionnaire values ranging from 1 “disagree strongly” to 5 “agree strongly” were categorised into the 5 dimensions of personality traits i.e., extraversion, openness, agreeableness, conscientiousness, and neuroticism and all 5–7 individual item values of each dimension added up to one score per participant and dimension. For specific items (question numbers 2,6,8,9,12,18,21,23,24,27,30,33,34,36,40,41) the values were converted to reverse-score values, thus reflecting the personality dimension accordingly [49]. Single missing values in the BFI-42 questionnaires were extrapolated according to the woman’s other stated values of said specific personality dimension to ensure usability of said personality dimension score in comparison with other individuals [49].

In a subgroup analysis, personality trait dimensions were compared in the vulvar pain groups (idiopathic vulvodynia, eczema and lichen groups) depending on symptom severity in terms of pain frequency, duration of pain, and feelings of vaginal tightness.

### Statistical methods

All data was analysed using a descriptive statistical approach according to the STROBE guidelines for cross-sectional studies. For this matter the programme SPSS version 29 was used for most calculations. Exclusively Fisher’s exact test and confounder control for age in the sexual violence/abuse analysis and Wilcoxon rank-sum tests with Holm correction in the personality analysis were conducted with R version 4.5.0. The imputation method used for single missing values in the BFI-42 questionnaire consisted of calculating mean scores of a participants stated other values in a personality dimension and multiplying said mean score by the factor of total number of items of said personality dimension.

## Results

Due to skewed distribution and, since sample sizes were limited, median and IQR measures were used instead of means and standard deviations.

### Patient characteristics

Of the 128 idiopathic vulvodynia, 117 eczema and 81 lichen patients as well as 191 asymptomatic women contacted, 34 idiopathic vulvodynia, 31 eczema and 20 lichen patients and 30 asymptomatic women engaged in the study, corresponding to response rates of 26.6%, 26.5%, 24.7% and 15.7%, respectively. The median age was highest in the lichen group (with median = 48years, IQR = 29, whereas median age was 33 to 35 years (IQR 11–12) in the idiopathic vulvodynia, eczema and the asymptomatic control groups, respectively (Table 1). Most of the women in all participant groups were European. 58.8% of all idiopathic vulvodynia patients possess a high school or university degree and overall all patient cohorts the idiopathic vulvodynia group had the highest percentage of an advanced education level.

**Table 1:** Socio-epidemiologic data of participants, (*n* = 115)

Participants, *n*	Idiopathic vulvodynia group (V)	Eczema group (E)	Lichen group (L)	Control group (C)
	34	31	20	30
Median (IQR) age in years	34.5 (12)	33 (11)	48 (29)	35 (11)
Ethnicity/nationality, n (%)				
European	32 (94.1%)	29 (93.5%)	20 (100%)	26 (86.7%)
Non-European	2 (5.9%)	2 (6.5%)	0 (0%)	4 (13.3%)
Education, n (%)				
Secondary school degree	10 (29.4%)	13 (41.9%)	7 (35%)	9 (30%)
High school/university degree	20 (58.8%)	12 (38.7%)	9 (45%)	14 (46.7%)
Other/not specified	4 (11.8%)	6 (19.4%)	4 (20%)	7 (23.3%)

### History of sexual violence/abuse experiences and clinical data

Table 2 gives an overview on sexual violence/abuse experiences and clinical data of vulvar pain patients. A quarter of all vulvar pain patients reported a history of sexual violence/abuse. The eczema group showed the highest overall percentage (35.5%), which was twice the sexual violence/abuse rate reported by idiopathic vulvodynia patients. In total, 17.7% of idiopathic women patients had a history of sexual violence/abuse and their vast majority stated solely emotionally hurtful sexual violence/abuse experiences without physical harm, in contrast to approximately half the eczema and lichen patients with a history of sexual violence/abuse, respectively. The prevalence of sexual violence did, however, not differ significantly between idiopathic vulvodynia, eczema and lichen patients (Fisher’s exact test, *p* = 0.276). Sensitivity analyses comparing idiopathic vulvodynia to eczema and lichen separately, yielded consistent non-significant results with wide confidence intervals (idiopathic vulvodynia vs. eczema: OR 2.44, 95% CI 0.69–9.50; *p* = 0.16, and idiopathic vulvodynia vs. lichen: OR 1.12, 95% CI 0.20–5.60; *p* = 1.00), indicating no robust group differences in this sample. For confounder control purposes, an age-adjusted logistic regression was conducted. Comparing idiopathic vulvodynia patients to the dermatologic vulvar pain groups, history of sexual violence was not significantly associated with idiopathic vulvodynia (adjusted OR 0.48, 95% CI 0.15–1.37; *p* = 0.18). Age was also not significantly associated with the outcome (OR per year 0.97, 95% CI 0.93–1.01; *p* = 0.11).

**Table 2:** Sexual violence/abuse experiences and clinical data of vulvar pain patients, (*n* = 85)

Participants, *n*	Idiopathic vulvodynia group (V)	Eczema group (E)	Lichen group (L)	*P*
	34	31	20	
Total history of sexual violence/abuse experiences, n (%)	6 (17.7%)	11 (35.5%)	4 (20%)	0.276^*1*^
History of sexual violence/abuse involving physical harm, n (%)	2 (5.9%)	5 (16.1%)	2 (10%)	
History of sexual violence/abuse without physical harm, n (%)	4 (11.8%)	6 (19.4%)	2 (10%)	
Symptom severity, median (IQR)				
Vaginal tightness 1 (never) – 3 (always)	2 (2)	2 (1)	2 (1)	
Pain frequency 1 (less than once a month) – 7 (more than once a day)	5 (2)	7 (4)	5 (3)	
Duration of pain 1 (seconds) – 5 (more than 3 days)	3 (2)	3 (3)	3 (3)	
Age of vulvar pain onset in years, median (IQR)	21 (14)	24.5 (15)	34 (31)	
No. of children born vaginally, n (%)				
0	27 (79.4%)	25 (80.6%)	12 (60%)	
1	5 (14.7%)	1 (3.2%)	3 (15%)	
2	2 (5.9%)	5 (16.1%)	3 (15%)	
≥ 3	-	-	2 (10%)	

^1^Calculated by Fisher’s exact test

The median value of vaginal tightness was 2, overall, for all patient groups, corresponding to “sometimes” experiencing a feeling of vaginal tightness when having intercourse/using a tampon. Median average of pain frequency was 5 in the idiopathic vulvodynia and lichen groups, corresponding to experienced pain several times a week, whereas it was 7 (more than once a day) in the eczema group. In all 3 vulvar pain groups, the median of average stated duration of pain was 3, corresponding to a pain lasting for “hours”. Median age of vulvar pain onset was 21 years in the idiopathic vulvodynia group, 24.5 years in the eczema and 34 years in the lichen group, respectively. Most vulvar pain patients had not given birth vaginally (79.4% of the idiopathic vulvodynia patients and 80.6% of the eczema patients), whereby with 40% of all lichen patients reporting 1 to 3 vaginal deliveries, the lichen group showed the highest number of vaginal births.

**Table 3:** Severity of symptoms in vulvar pain patients depending on history (SA) or absent history (nSA) of sexual violence/abuse, (*n* = 85)

Participants, *n*	Idiopathic vulvodynia group (V), 34		Eczema group (E), 31		Lichen group (L), 20	
	V (SA), 6	V (nSA), 28	E (SA), 11	E (nSA), 20	L (SA), 4	L (nSA), 16
Vaginal tightness 1 (never) – 3 (always) median (IQR)	2 (2)	2 (2)	2 (1)	2 (1)	2^*1*^	2 (1)
Pain frequency 1 (less than once a month) – 7 (more than once a day), median (IQR)	5 (3)	4.5 (3)	6 (2)	7 (4)	3^*1*^	5 (2)
Duration of pain 1 (seconds) – 5 (more than 3 days), median (IQR)	3 (3)	3 (2)	4 (3)	3 (3)	4^*1*^	3 (3)

^1^In subgroups with only 4 and overlapping responses no IQR was calculated

### Symptom severity in regard to experienced sexual violence/abuse

Overall, all median values of occurrence of vaginal tightness were corresponding to sometimes experiencing vaginal tightness when having intercourse/using a tampon, independent of reported history of sexual violence/abuse (Table 3). In the idiopathic vulvodynia group, median pain frequency was several times a week for patients with history of sexual abuse and once to several times a week for those without such a history. Median pain frequency was highest in the eczema group without history of sexual violence/abuse and lowest in the lichen group with stated history of sexual violence/abuse. In terms of duration of pain, the median measures of idiopathic vulvodynia patient with present and absent history of sexual abuse were for both groups equivalent to hours. The median duration of pain was highest in eczema and lichen patients that had endured sexual violence/abuse, whereas median duration of pain in the vulvodynia group was the same in the groups with and without sexual violence/abuse experience.

### Personality trait dimensions

All in all, 10 single item values were missing in the BFI-42 questionnaires of all 115 participants (max. 1 item per dimension and participant). The single missing values were therefore imputed according to the woman’s mean score of the other reported values of said specific personality dimension.

Table 4 shows median values and IQR of all 5 personality dimensions in the idiopathic vulvodynia, eczema, lichen and asymptomatic control groups. Median values of extraversion were lowest in the lichen and highest in the asymptomatic control group, whereas the median values of agreeableness were similar in all participant groups (17 in the idiopathic vulvodynia and lichen groups) and 18 the eczema and asymptomatic control groups, respectively. The eczema group showed the highest median value in conscientiousness and the lowest in neuroticism, whereas the lichen group scored the highest median in neuroticism. Median values of openness were highest in the eczema and lowest in the control group.

**Table 4:** Big Five personality trait analysis in different participant groups, (*n* = 115)

Participants, *n*	Idiopathic vulvodynia group (V), 34	Eczema group (E), 31	Lichen group (L), 20	Control group (C), 30
Extraversion, median (IQR)	21 (7)	20 (7)	17.5 (7)	22 (7)
Agreeableness, median (IQR)	17 (5)	18 (6)	17 (4)	18 (4)
Conscientiousness, median (IQR)	18 (5)	20 (7)	18 (6)	18 (5)
Neuroticism, median (IQR)	21 (7)	19 (7)	22.5 (7)	21 (6)
Openness, median (IQR)	22 (8)	24 (8)	22 (7)	21 (12)

As demonstrated in Table 5, none of the Big Five personality dimensions differed significantly in any comparison when comparing idiopathic vulvodynia separately to eczema, lichen, and asymptomatic controls (Wilcoxon rank-sum tests with Holm correction for multiple testing). Effect sizes were consistently small across traits and comparisons (Cliff’s delta values close to zero with confidence intervals crossing zero), indicating no clear personality-based differences between vulvodynia and the respective comparison groups in our sample.

**Table 5:** Comparison of Big Five personality traits between idiopathic vulvodynia (V), eczema (E), lichen (L) and control groups (C), (*n* = 115)

Group comparison	Trait	*n* (V / comparison group)	median V	median comparison group	*p*	*p* (holm-correction, 5 traits)^1^	Cliff’s 𝛿 [95% CI]
V vs. C	Extraversion	34 / 30	18	22	0.173	0.864	-0.2 [-0.46, 0.09]
V vs. C	Agreeableness	34 / 30	17	18	0.400	1.000	-0.12 [-0.38, 0.16]
V vs. C	Conscientiousness	34 / 30	18	18	0.973	1.000	0.01 [-0.27, 0.28]
V vs. C	Neuroticism	34 / 30	21	21	0.957	1.000	-0.01 [-0.28, 0.27]
V vs. C	Openness	34 / 30	22	21	0.558	1.000	0.09 [-0.18, 0.34]
V vs. E	Extraversion	34 / 31	18	20	0.36	1.000	-0.13 [-0.4, 0.15]
V vs. E	Agreeableness	34 / 31	17	18	0.673	1.000	-0.06 [-0.32, 0.21]
V vs. E	Conscientiousness	34 / 31	18	20	0.381	1.000	-0.13 [-0.39, 0.16]
V vs. E	Neuroticism	34 / 31	21	19	0.123	0.615	0.22 [-0.06, 0.48]
V vs. E	Openness	34 / 31	22	24	0.937	1.000	0.01 [-0.26, 0.29]
V vs. L	Extraversion	34 / 20	18	17.5	0.673	1.000	0.07 [-0.23, 0.36]
V vs. L	Agreeableness	34 / 20	17	17	0.928	1.000	-0.02 [-0.32, 0.29]
V vs. L	Conscientiousness	34 / 20	18	18	0.547	1.000	0.1 [-0.22, 0.4]
V vs. L	Neuroticism	34 / 20	21	22.5	0.753	1.000	-0.05 [-0.36, 0.27]
V vs. L	Openness	34 / 20	22	22	0.627	1.000	0.08 [-0.24, 0.39]

^1^Holm-correction was used due to multiple testing

Comparing personality traits in variously affected vulvar pain patient groups, median neuroticism scores were highest in the least affected patient cohorts concerning pain frequency and duration of pain overall all 3 vulvar pain groups (Table 6). Median neuroticism was also highest in eczema and lichen patients that never experienced vaginal tightness during sexual intercourse or use of a tampon, yet, highest in the idiopathic vulvodynia subgroup that sometimes experienced vaginal tightness.

Overall, the highest medians of extraversion values were stated in the eczema group (medium affected subgroup concerning duration of pain and least affected subgroups concerning pain frequency and vaginal tightness). The highest scores of median agreeableness were stated in the idiopathic vulvodynia subgroup with high pain frequency and lichen subgroup with medium duration of pain and the lowest in the eczema subgroup with medium duration of pain, respectively. The highest median conscientiousness scores were measured in the eczema subgroups suffering from low/medium pain frequency and low duration of pain, yet also the eczema subgroup most often experiencing vaginal tightness. Median openness values were highest in the lichen subgroups that stated low and high pain frequency and medium duration of pain as well as eczema patients reporting medium and high pain frequency and medium and long duration of pain as much as vaginal tightness during every intercourse/use of a tampon.

**Table 6:** Big Five personality trait subgroup analysis in vulvar pain patients, (*n* = 85)

Participants, (*n*)	Idiopathic vulvodynia group (*n* = 34)			Eczema group (*n* = 31)			Lichen group (*n* = 20)		
Pain frequency^*1*^, (n) according to symptom severity	Low (*n* = 2)	Medium (*n* = 22)	High (*n* = 7)	Low (*n* = 2)	Medium (*n* = 11)	High (*n* = 17)	Low (*n* = 2)	Medium (*n* = 11)	High (*n* = 4)
Personality trait dimensions median, (IQR)									
Extraversion	17.5^*3*^	18 (6)	16 (14)	21.5^*3*^	20 (7)	18 (8)	18^*3*^	16 (5)	18 (16)
Agreeableness	18.5^*3*^	16 (6)	19 (6)	17.5^*3*^	18 (7)	17 (7)	17.5^*3*^	17 (7)	16.5 (1)
Conscientiousness	17.5^*3*^	17.5 (7)	18 (4)	22.5^*3*^	23 (7)	19 (8)	21^*3*^	18 (5)	15 (5)
Neuroticism	24.5^*3*^	21 (6)	19 (8)	21.5^*3*^	18 (6)	19 (7)	25.5^*3*^	22 (9)	21.5 (7)
Openness	21^*3*^	23 (12)	22 (1)	17^*3*^	24 (7)	25 (12)	25.5^*3*^	20 (10)	25 (8)

Duration of pain^*2*^, (n) according to symptom severity	Short (*n* = 13)	Medium (*n* = 11)	Long (*n* = 9)	Short (*n* = 9)	Medium (*n* = 6)	Long (*n* = 14)	Short (*n* = 7)	Medium (*n* = 4)	Long (*n* = 8)
Personality trait dimensions median, (IQR)									
Extraversion	18 (9)	18 (5)	17 (10)	18 (9)	22 (7)	20.5 (6)	16 (6)	14.5 (10)	19.5 (11)
Agreeableness	17 (7)	17 (7)	16 (4)	18 (5)	12.5 (8)	18 (4)	17 (4)	19.5 (7)	14.5 (4)
Conscientiousness	17 (6)	19 (7)	18 (8)	24 (9)	16.5 (6)	21 (6)	19 (2)	16 (9)	16 (8)
Neuroticism	24 (8)	19 (7)	20 (6)	20 (7)	18 (6)	18 (7)	23 (5)	21 (12)	22.5 (7)
Openness	22 (11)	21 (10)	22 (8)	18 (9)	26 (10)	24.5 (9)	22 (7)	24 (10)	20.5 (11)

Vaginal tightness, (n) according to symptom severity	Never (*n* = 12)	Some-times (*n* = 7)	Always (*n* = 15)	Never (*n* = 12)	Some-times (*n* = 13)	Always (*n* = 5)	Never (*n* = 5)	Some-times (*n* = 9)	Always (*n* = 4)
Personality trait dimensions median, (IQR)									
Extraversion	17 (5)	20 (12)	18 (8)	21 (6)	18 (8)	20 (11)	19 (11)	16 (8)	17 (17)
Agreeableness	16.5 (6)	17 (5)	17 (5)	17 (6)	18 (7)	18 (12)	17 (7)	17 (6)	16 (3)
Conscientiousness	16.5 (5)	21 (7)	18 (5)	20.5 (8)	20 (9)	23 (8)	17 (7)	19 (8)	18 (2)
Neuroticism	21 (8)	24 (3)	18 (7)	21.5 (8)	19 (5)	17 (9)	23 (12)	22 (5)	20 (8)
Openness	22 (7)	22 (4)	22 (14)	25 (6)	20 (11)	24 (9)	20 (3)	22 (15)	23 (9)

Big Five personality trait subgroup analysis in association with varying pain frequency, duration of pain and vaginal tightness in idiopathic vulvodynia, eczema and lichen patients

^1^Pain frequency: Vulvar pain once a month or less = low, > once a month - once a week = medium, at least once a day = high

^2^Duration of pain: Seconds up to minutes = short, hours = medium, days = long

^3^In subgroups with less than 4 responses, no IQR was calculated

## Discussion

In our collective of idiopathic vulvodynia patients and eczema and lichen patients the sexual violence/abuse rate was higher than estimated in the general female population in Europe [36], which is consistent with previous literature [18, 42, 51]. This outcome is in line with other gynecological and obstetrical diseases and complications, which show increased prevalence in women with sexual abuse experiences [52–55]. Moreover, sexual abuse victims are prone to sexual dysfunction, dyspareunia and lower relationship quality [56–58]. And furthermore, somatic dysfunctions including chronic fatigue, sleeping disorders and heart and bladder issues have also been associated with sexual abuse [28, 29]. However, with the lowest sexual violence/abuse rate found in the idiopathic vulvodynia group in comparison with the dermatologically derived vulvar pain groups, the present study did not support our hypothesis of previous sexual violence/abuse experiences to be a particular associated factor in idiopathic vulvodynia. Furthermore, our findings concerning symptom severity in a subgroup analysis, examined in terms of pain frequency, duration of pain and a feeling of vaginal tightness during intercourse or tampon use, did not indicate a variation in idiopathic vulvodynia patients regarding present or absent history of sexual violence/abuse. Against our expectations, overall all vulvar pain groups median pain frequency was highest in the eczema group without history of sexual violence/abuse and lowest in the lichen group with present history of sexual violence/abuse; although, median duration of pain was highest in eczema and lichen patients that had endured sexual violence/abuse. However, it is important to view these findings in context of the limited sample size, as a relevant association between sexual violence/abuse and idiopathic vulvodynia as much as symptom severity in this and/or all vulvar pain groups might only be visible in larger study cohorts. In addition, current research indicates that both number and type of emotional events such as abuse can influence pain expression in pain syndromes such as idiopathic vulvodynia [17]. Therefore, an analysis of vulvar pain symptoms in idiopathic vulvodynia patients regarding timing and extent of sexual violence/abuse could be of substantial relevance.

Furthermore, recent studies including a meta-analysis emphasised frequent co-occurrence of idiopathic vulvodynia with depression and anxiety [23, 25]; psychosomatic factors considered as significant in nociplastic pain syndromes that have not been included in our present research. As previous treatment approaches have not been analysed in the present study, it cannot be excluded that treatment status (e.g. previous psychotherapeutic interventions) may have acted as a confounder regarding symptom severity perception and should be taken into account in future multivariate analysis. Hormonal imbalances might also influence vulvar pain, since genital pain has been affirmed to change across the menstrual cycle [59]. As age is also associated with diverging hormonal status, the lichen group (with a median age that was 13.5 years higher than the respective median in the idiopathic vulvodynia group) would not have provided an eligible control group in this matter. Furthermore, even though the vast majority of all women included in the study were European, potential cultural differences of pain perception have not been analysed in our study.

The assumption of a certain personality profile in idiopathic vulvodynia patients that might either be a precursor or consequence of vulvar pain could not be substantiated in the present study. Additionally, previously stated personality differences in vulvar pain patients compared to the general population could not be reaffirmed [21, 22, 41]. Patients with neuroticism, associated with rumination as an inadequate form of emotional regulation, have been found to experience an enhanced response to pain stimuli and been associated with poor coping in stressful situations [60, 61]. Suso-Ribera et al. have also recently established a significant correlation of neuroticism and pain severity [40]. In our study, however, idiopathic vulvodynia patients as well as the other vulvar pain subgroups reporting the highest pain frequency and longest duration of pain levels showed the lowest self-reported neuroticism scores. Idiopathic vulvodynia patients with a tendency to neuroticism might, however, have sought treatment at an earlier stage, thereby alleviating the symptom burden of the chronic condition. In addition, due to a lack of a gold standard treatment for idiopathic vulvodynia patients, this specific patient cohort is likely to have undergone a psychotherapeutic treatment approach more frequently than eczema and lichen patients. With psychosomatic treatment approaches in idiopathic vulvodynia patients showing promising treatment results in substantial symptom alleviation, possibly also the negative perception of physical and mental health and rumination, that are associated with neurotic personality traits, might have been addressed and influenced. This possible explanation is in line with research suggesting neuroticism as a key factor in psychological treatment approach [40]. Furthermore, referral of the idiopathic vulvodynia patients that have been examined in this study to the specialised vulva clinic might have been a positive experience for the patients dealing with the disease in terms of feeling that their complaints are taken seriously. Particularly in patients with high levels of neuroticism, this might have led to reduction in rumination. In our study, idiopathic vulvodynia patients had the highest prevalence of advanced educational level, a finding that is consistent with recent research suggesting a high education level in idiopathic vulvodynia patients and, thus, a possibly more pronounced ambitious personality trait [8]. A substantial difference in the personality dimension “conscientiousness” that includes ambition of idiopathic vulvodynia patients in comparison to the other vulvar pain groups could, however, not be ascertained in the present analysis. With a remarkably low response rate in study participation overall all groups investigated, this matter might be explained by a non-response bias due to less conscientious women less frequently engaging in such a study.

### Strengths and limitations

This is to our knowledge the first study to evaluate associated psychosomatic factors in idiopathic vulvodynia patients compared to somatically attributable vulvar pain patients and only the third study investigating a highly selective idiopathic patient cohort screened thoroughly for underlying somatic explanations.

Limitations include the small sample size, thus limiting statistical power significantly. Statistical analyses were therefore of exploratory character and primarily aimed at effect estimation rather than hypothesis testing. Only moderate-to-large differences would have been detectable, and smaller but potentially relevant effects cannot be ruled out. The matter of limited participation might be explained by most patients’ last visit in the vulva clinic dating back months to years before study conduct and/or by meanwhile improved symptoms and therefore reduced interest. The overall low response rate further increases the risk of non-response and self-selection bias. With 15.7%, the response rate in the control group was particularly low, yet, this is a known phenomenon in various medical fields, comparable to those of other studies [62]. On the other hand, the participants that had engaged in the study did fill out most of the genital pain questionnaire and over 95% of the BFI-42 questionnaire. Overall, only 3 participants (2 idiopathic vulvodynia and 1 eczema patient) had to be excluded due to incomplete data (< 50% of items answered in BFI-42 questionnaire). Thus, in the personality analysis only 10 of the overall 4830 items (42 question items times 115 participants) were imputed, resulting in a negligible potential bias. On the other hand, BFI-42 questionnaire is a sole self-evaluation concerning personality traits without a simultaneous objective perception.

Also, since the control group did not fill out the genital pain questionnaire, we lacked information concerning sexual violence/abuse experiences in this cohort as a relevant limitation. In addition, details of the sexual violence/abuse experienced by affected vulvar pain patients (e.g. timing, severity and recurrence) were not examined. Since potential sexual violence/abuse was addressed exclusively in the form of an online questionnaire and intrusive images in traumatised patients are often triggered by rumination [63], the authors carefully avoided detailed questioning about sexual violence/abuse history in this setting without professional guidance. Yet, the lack of this information impeded further analysis, e.g. a comparison of the extent and/or the patient’s age at (first) sexual violence/abuse experience and stated symptom severity of their vulvar pain. Also, psychiatric comorbidities such as depression, anxiety and posttraumatic stress as well as chronic pain disorders and prior treatment history were not integrated in this study and might have influenced pain or personality self-evaluation.

In addition, the lichen group consisted of 3 different lichen subgroups and the median age of lichen patients was 48 years in contrast to the median age of 33 to 35 years in the other participants groups. This age gap is considered as a relevant limitation by the authors since personality scores, sexual violence/abuse experience and pain perception have been indicated to change with age [50, 64, 65]. Lastly, obstetric injury history was not examined and due to the single-center study design a referral bias is possible.

### Conclusions and clinical relevance

In this study, higher sexual abuse rates among women with idiopathic vulvodynia and somatically derived vulvar pain patients vs. the general female population supports the role of sexual violence/abuse experiences as an associated factor in the disease. With the lowest prevalence of sexual violence/abuse history in idiopathic vulvodynia patients, such experiences could not, however, be affirmed as a specific factor in idiopathic vulvodynia and given the cross-sectional study design our findings describe associations rather than causal relationships. Also, no remarkable variation could be assessed in symptom severity of vulvodynia patients regarding history of sexual violence/abuse. Similarly, our findings did not suggest a particular personality profile (e.g. higher levels of neuroticism) in idiopathic vulvodynia patients. Also, patient groups with the highest symptom severity levels showed the lowest scores in neuroticism, yet, frequently used psychosomatic therapy approaches in idiopathic vulvodynia patients might have positively influenced pathologically enhanced neurotic personality features.

In conclusion, further research on associated psychosomatic factors and comorbidities in the specific patient cohort of idiopathic vulvodynia is therefore essential. We advise clinicians supporting women with vulvodynia to be aware of the considerable prevalence of sexual violence/abuse experiences in this patient cohort and to evaluate comprehensive multidimensional treatment, including psychological interventions.

## Data Availability

The datasets generated and/or analysed during the current study are not publicly available due to possibly compromised individual privacy regarding the limited data but are available from Co-author Prof. Dr. med. Cornelia Betschart on reasonable request.
